# Microorganism Response to Stressed Terrestrial Environments: A Raman Spectroscopic Perspective of Extremophilic Life Strategies

**DOI:** 10.3390/life3010276

**Published:** 2013-03-13

**Authors:** Susana E. Jorge-Villar, Howell G.M. Edwards

**Affiliations:** 1 Area Geodinamica Interna, Facultad de Humanidades y Educacion, Universidad de Burgos, Calle Villadiego, Burgos 9001, Spain; 2 CENIEH, P° Sierra de Atapuerca, s/n, Burgos 09002, Spain; 3 Centre for Astrobiology and Extremophiles Research, School of Life Sciences, University of Bradford, Bradford BD7 1DP, UK; E-Mail: h.g.m.edwards@bradford.ac.uk; 4 Space Research Centre, Department of Physics & Astronomy, University of Leicester, Leicester LE1 7RH, UK

**Keywords:** Raman spectroscopy, extremophile, bio-strategy, geo-strategy, microorganism

## Abstract

Raman spectroscopy is a valuable analytical technique for the identification of biomolecules and minerals in natural samples, which involves little or minimal sample manipulation. In this paper, we evaluate the advantages and disadvantages of this technique applied to the study of extremophiles. Furthermore, we provide a review of the results published, up to the present point in time, of the bio- and geo-strategies adopted by different types of extremophile colonies of microorganisms. We also show the characteristic Raman signatures for the identification of pigments and minerals, which appear in those complex samples.

## 1. Introduction

Since the first reports in the literature of the Raman spectroscopic analyses of the biological colonization of toxic mineral pigments in Renaissance frescoes [1] in 1991 and that of an endolithic colonization of Beacon sandstone from the Antarctic peninsula [2,3] in 1997, the analytical interrogation of extremophilic systems with a variety of geological substrates has resulted in novel information being provided about the nature of the synthetic protective chemicals and the strategies being employed for survival of the colonies in terrestrial stressed environments using this technique. At first, the identification of the chemical complexes formed as a result of the reaction of lichen metabolic waste products upon calcareous substrates in the form of oxalates was a primary objective [4,5,6,7,8] and it was not until much later that the characterization of the organic by-products was accomplished through the adoption of longer wavelength laser excitation and comparison with extracted materials [9,10,11,12,13,14]. The successful survival of extremophiles in stressed environments is dependent on their adaptation to the prevailing conditions of high or low temperatures, extreme desiccation, high energy ultraviolet insolation, high or low barometric pressures, extremes of pH in the range of <1 to >12 and on the presence of toxic chemicals and ions such as mercury (II), antimony (III), lead (II), barium (II), arsenic (III) and copper (II), which are found in many minerals. It was also apparent that the production of protective chemicals by extremophiles as a response to environmental stresses was supported by their ability to adapt their geological matrices and substrates, often resulting in biogeological signatures, which have remained in the geological record long after the extinction of the biological colonies.

It was appreciated at the outset that a major advantage of Raman spectroscopy as an analytical technique for the study of extremophilic colonization and survival was its ability to interrogate a system microscopically across a horizontal or vertical transect without any physical separation or chemical and mechanical pretreatment such as coating, grinding or polishing [15,16,17,18]. Hence, information about the interaction between the biological and geological components is accessible naturally without extraction or modification of the biogeological system. This was appreciated in the analysis of works of art, which had been subjected to lichen deterioration and required conservation [19,20,21,22].

The recognition of key definitive Raman spectral signatures in the same spectrum from the organic and inorganic components is a desirable outcome for the projected deployment of a miniaturized spectrometer unit as part of the life detection instrumentation suite on the planetary rover vehicle on the ESA ExoMars mission scheduled for 2018. In this mission, the Raman spectrometer will be a first-pass probe of selected samples removed from the Martian surface and subsurface (down to a depth of two meters) for the detection of extremophilic spectral signatures from extinct or extant colonies in niche environments. The assimilation of Raman spectral data and signatures into a database relating to extremophiles from terrestrial Mars analogue sites is therefore of vital importance, and this has been undertaken in recent years and is still ongoing [23,24,25,26,27,28,29,30]. Similarly, Raman spectroscopic analyses of other extreme terrestrial sites, which have relevance to the remote exploration of other planets and their satellites in our Solar System have been reported, such as glaciers, deep-sea smokers, hot geysers and snowfields, which have an ambience with planetary icy moons such as Europa, Titan, Io and Enceladus that are also noteworthy in this respect.

The purpose of the present paper is to survey and to critically examine comprehensively the existing wealth of Raman spectral data and their interpretation for terrestrial extremophiles; from this analysis, which has never been undertaken comprehensively hitherto, the following deductions can then be made:

- the commonality of Raman spectral signatures between various sites and extreme environments;
- the factors which affect the recording of Raman spectral signatures from the extremophilic colonization of terrestrial sites;
- the definition of Raman data for evidence of extinct or extant life in the geological record.

## 2. Results and Discussion

### 2.1. Technical Considerations of the Use of Raman Spectroscopy for the Study of Extremophiles

Raman spectroscopy is an analytical technique, which provides molecular information for either organic or inorganic compounds [31]. The Raman effect allows one to probe the internal structure and bonds between atoms in a molecule.

**Figure 1:**
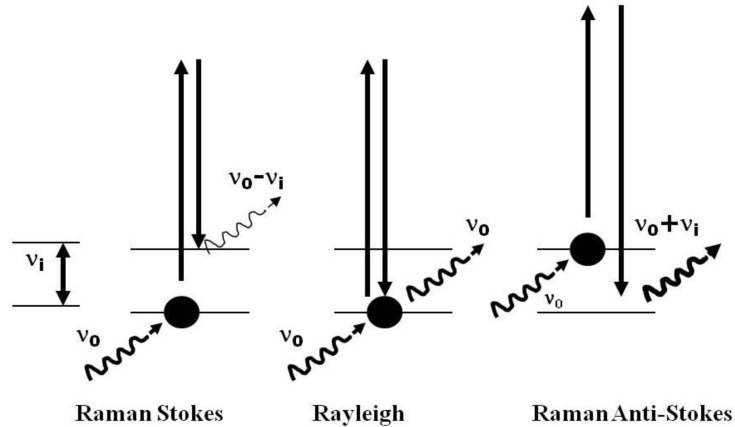
Molecular light scattering; Raman Stokes, the molecule absorbs energy; Rayleigh: there is neither absorption nor loss of energy; Raman anti-Stokes: the molecule loses energy.

When monochromatic laser radiation falls upon a given compound, molecules can jump to a higher energy level (Figure 1); most of these molecules fall back to the same energy level, with the emission of a photon that has the same frequency as the incident light leading to Rayleigh scattering. In contrast, some molecules, which are already in higher or lower energy levels, can either emit or absorb a photon upon irradiation and then make a transition to lower or higher energy levels giving rise to Stokes and anti-Stokes Raman radiation. The result is a spectrum which is characteristic of each molecule and which exhibits a definitive number of key spectral signatures at characteristic wavenumbers, so providing a molecular fingerprint that is analytically useful for molecular species characterization.

The use of Raman spectroscopy for the study of extremophiles has some advantages with regard to other techniques. No sample preparation, neither chemical nor physical, is required, and further no dilution, concentration, staining, grinding, or desiccation is needed, so the technique is non destructive. Of course, it is necessary to expose the surface for analysis, and, nowadays, with portable instruments, samples can be analyzed in the field nondestructively, a major advantage for further analytical procedures to be undertaken with micro- and macro- analyses made possible by using different sample illumination or collection lenses. Furthermore, sample size is not a problem, as a microscope can be used for the interrogation of small sample regions and an optical fiber head can be attached and used to study very large specimens. Moreover, the recent development of portable instruments allows the in-field use of Raman spectrometers under environmental conditions, which obviates changes that may occur to the sample during the acquisition of samples from the field and their transportation and subsequent storage prior to spectroscopic analysis.

However, although Raman spectroscopy is a very useful technique, it has some disadvantages. Some compounds give a strong fluorescence emission, which can be several orders of magnitude greater than the Raman signal and masks the inherently weaker Raman bands. As this technique is based upon the use of a laser, usually operating in the infrared or visible region of the electromagnetic spectrum, absorption of the radiation can induce sample heating, which can result in molecular or mineralogical changes or, in the worst-case scenario, even sample degradation or burning. It is clear then that the laser power used for spectral acquisition should be used with the lowest power possible consistent with the acquisition of the spectrum even though this may require correspondingly greater spectral accumulation times. Furthermore, the laser wavelength used for exciting the sample can influence the fluorescence emission and also the relative intensity of the Raman bands: Hence, a strong signature observed using a particular laser wavelength can become only of medium or weak intensity when an alternative laser wavelength is used. This effect can cause problems in spectrum interpretation.

A particular difficulty lies in the nature of the extremophiles themselves. These are organisms usually living in or on various supports, ranging from ice to stone, soil, rocks, wood, and synthetic substrates such as plastics, glass, metals and ceramics. There is a complex mixture of signatures from organic and inorganic compounds arising from the extremophiles themselves and from their substrates that can be observed in the Raman spectrum. Each compound will show a variable number of Raman signatures in admixture with those from other compounds, and the observed intensity of the significant Raman bands for any given compound in the spectrum is related directly to the proportional amount of the compound in the sample. A further complication is that the range of molecular scattering factors for different species in the Raman effect confers a different response of the molecular material to its interaction with the incident laser radiation, and this can additionally dependent upon the laser wavelength; an example of this is the intensity enhancement of carotenoid signatures in extremophile spectra excited using a green or blue laser ascribed to resonance Raman scattering processes.

The critical functionality region for organic molecules lies in the range between 1,800 to 1,000 cm^−1^ in the Raman spectrum, whereas for minerals, this is most characteristic in the 1,100 to 100 cm^−1^ region. Minerals do not usually show bands at higher wavenumbers than this, with the exception of water and hydroxyl bands; spectral bands in the 3,000–3,500 cm^−1^ region are characteristic of coordinated water and OH groups; in the lower spectral wavenumber region below 1,000 cm^−1^, the Raman spectral signatures of both organic and inorganic compounds occur together.

When the Raman spectrum of an extremophilic organism shows the spectrum of a mixture of possible compounds, either organic and/or mineral, the superposition and overlapping of Raman signatures is realizable and, then, individual bands become broader and a peak band envelope can be observed to move to lower or higher wavenumbers. In this case, comparison with the database for molecular characterization based on pure materials is fraught with difficulty and care must be taken in the interpretation of the spectral data.

Another important consideration for the spectral interpretation lies in the fact that the Raman database of biomolecules from natural samples is rather limited and is naturally based upon detailed spectra from extracted and purified materials; hence, it is not always possible to assign all bands to a particular compound in a complex mixture unless characteristic features of each species are known and several features necessarily remain tentative or unassigned. More supporting studies linked to the identification of components extracted from extremophiles need to be carried out in the future for enlarging the Raman database and facilitating the unambiguous identification of biomolecular signatures in complex systems.

Extremophiles are described as those organisms able to live and survive under extreme conditions such as desiccation, extremes of temperature, pressure, pH, high salinity, radiation insolation and restricted nutrient and oxygen availability [32,33,34,35]. Most of these organisms are unicellular, mainly prokaryotics, although bacteria are also widespread [32]. There is a general idea that organisms living under similar hazardous environmental conditions develop similar adaptive survival strategies. Those strategies necessarily combine biological and geological mechanisms, such as the production of pigments, changes in cellular membranes (bio-strategies) or migration into the rock substratum and mineralogical alterations (geo-strategies) in their adaptation strategies for successful survival under hostile environments [33,36,37,38,39].

The analyses of extreme organisms using Raman spectroscopy started first in the 1990s. Up to the present time, most of the specimens studied have been related to cold and hot deserts, where the availability of water, together with extreme temperatures are the main restrictive environmental parameters (Table 1)—see Table 1 for references. No work, to our knowledge, has been undertaken on the characterization of extremophilic survival strategies without extraction using Raman spectroscopy for lack of oxygen or high pH habitats. For high pressure in the deep ocean environments, papers focus on the identification of minerals and sulfur, sometimes related to biological activity [40,41,42]. For high levels of cosmic radiation [43], only one paper has been published with the aim of detecting the degradation of biosignatures by Raman spectroscopy. In acid environments [44], only one paper, to our knowledge, has addressed the residual organic signatures in rocks.

The interest in the study of extremophiles rests mainly on the potential use of these organisms in industrial biotechnology and medical fields and in extraterrestrial life signature characterization. The search for life beyond the Earth has encouraged the survey of terrestrial extremophilic strategies, and most of the studies on extremophiles using Raman spectroscopy have been carried out with this purpose. The capabilities of Raman spectroscopy as an analytical technique have led it to be adopted as a part of the instrumental suite to be sent to Mars in the ExoMars-C 2018 mission led by the European Space Agency (ESA) [45].

### 2.2. Analysis of the Data

Although a large number of relevant papers have been surveyed for this review, many of them are not specifically linked to the stated theme because they analyze extremophiles grown under laboratory conditions [46,47]. Other studies do not identify compounds but specific signatures related to generically important but nonspecific organic biomaterials, such as those characteristic of some proteins, aminoacids, fatty acids, *etc* [47,48]; in other articles related to the study of extremophiles, the use of Raman spectroscopy was directed towards mineral identification [49,50], or only carbon was identified as organic compound and other analytical techniques were utilized for the organic molecule characterization [51]. Studies of bacterial specimens have also been carried out using surface enhanced Raman spectroscopy (SERS) [52,53,54], a Raman application in which the signal is enhanced by using a specially designed colloidal silver or gold substrate over which the sample is fixed by adsorption; in this case, as sample preparation is required, the analytical method is not strictly non-destructive. Although several of these analyses have studied extremophiles, it should be noted that extraction of the relevant organic compounds or sample manipulation was required for the analyses [55,56,57].

The analyses of the published papers reported here are related to conventional Raman spectroscopy of naturally occurring extremophilic microorganisms, in which the molecular characterization of the spectral signatures was accomplished without sample manipulation; these are summarized in Table 1, Table 2. References to the research works are given in Table 1 [58,59,60,61,62,63,64,65,66,67,68,69,70,71,72,73,74,75,76].

Table 1 shows the specimens related to origin/climate, environmental conditions and their substratum. Most of the samples came from the cold deserts of Antarctica and the Arctic, specifically from Svalbard (samples SP1A, SP1B, SP2 and SP10) or the north of Canada (SP7). 

Extremophiles may be characterized as epiliths, chasmoliths or endoliths, depending on whether the organisms are surface dwelling, have colonized cracks in rocks or, finally, have created subsurface colonies (geostrategies) within the pores of rocks. Usually, endoliths create a colored band a few millimeters below the surface of porous rocks. Although we do not present here a statistical study giving the range of endoliths analyzed, we should note that the most frequent endolithic rock in this review is a sandstone. This is believed to reflect the characteristics of this rock, since it has very well connected pores, which enable air and water to move easily across the rock transect. Another interesting characteristic is that it is composed of quartz, a translucent mineral that allows sunlight to reach the subsurface colony and initiate the chlorophyll function. 

Light penetration is a limiting factor for this chlorophyll function in the case of organisms living inside a rock (either in pores, cracks or vacuoles). Organisms have solved this problem either by living as close to the surface as possible or through the production of complementary light-harvesting pigments, such as c-phycocyanin, which are accessory pigments to chlorophyll for the harnessing of photoactive radiation (bio-strategies). In some cases, mineralogical changes as well as mineral mobilization (geo-strategies), directly related with the colonized layer, have been noticed. It is possible to observe these physical changes in the system from the Raman spectroscopic data and relate them to to a biogeological strategy.

**Table 1:** Extremophile specimens and related substratum (End = endolith; Chas = chasmolith; Epi = epilith; Sand = sandstone; MR = magmatic rock; Mag = Magnesite; Dol = dolomite; Mar = marble; Gyp = gypsum; Apl = aplite; Fum = fumarole; LB = lava basalt; bact mats = bacteria mats). The numbers in superscript in each specimen indicate the reference paper.

Specimen	Origin/Climate	Environmental Conditions	End	Chas	Epi	Sand	MR	Mag	Dol	Mar	Gyp	Salt	Apl	Fum	LB
ANT1 [58]	Antarctic		X			X									
ANT2 [58]	Antarctic		X			X									
ANT5 [58]	Antarctic		X			X									
EN1 [58]	Antarctic		X			X									
EN2 [58]	Antarctic		X			X									
RW [58]	Antarctic		X			X									
SP1A [59]	Arctic				X				X	X					
SP1B [59]	Arctic			X					X	X					
SP1C [59]	Tropical	Primary colonization			X									X	
SP2A [60]	Arctic			X		X									
SP2B [60]	Arctic			X		X									
SP2C [60]	Arctic		X			X									
SP2D [60]	Arctic		X			X									
SP2E [60]	Arctic				X	X									
SP3A [61]	Antarctic			X									X		
SP3B [61]	Antarctic			X									X		
SP4A [62]	Hot desert	Primary colonization			X	X									
SP4B [62]	Hot desert	Primary colonization			X		X								
SP5A [63]	Antarctic		X			X									
SP5B [63]	Antarctic	Exposed mats													
SP6A [64]			X					X							
SP7A [65]	Arctic	Halotrophic	X								X				
SP7B [65]	Arctic	Halotrophic	X								X				
SP8A [66]	Antarctic	Sediments													
SP9A [67]	Arctic	High temperature/ Biofilm													
SP10A [68]	Arctic			X											X
SP10B [68]	Arctic		X												X
SP11A [69]	Hot desert	Halotrophic/ Natron	X									X			
SP12A [70]	Hot desert	Halotrophic/ Halite			X							X			
SP12B [70]	Hot desert	Halotrophic/ Halite	X									X			
SP12C [70]	Hot desert	Halotrophic/ Halite	X									X			
SP12D [70]	Hot desert	Halotrophic/ Halite	X									X			
SP13A [71]	Antarctic	Snow algae													
SP13B [71]	Antarctic	Snow algae													
SP14A [72]	Antarctic		X			X									
SP14B [72]	Antarctic		X			X									
SP14C [72]	Antarctic		X			X									
SP15A [73]	Hot desert	Halotrophic	X									X			
SP15B [73]	Hot desert	Halotrophic	X								X	X			
SP15C [73]	Hot desert	Halotrophic/bact mats													
SP16A [74]	Antarctic			X					X	X					
SP17A [75]		Deep see vents/mats													
SP18A [76]	Hot desert	Halotrophic	X									X			
SP18B [76]	Hot desert	Halotrophic	X									X			
SP18C [76]	Hot desert	Halotrophic	X									X			

**Table 2:** Extremophile specimens and their strategies detected by Raman spectroscopy.

	ANT1	ANT2	ANT5	EN1	EN2	RW	SP1A	SP1B	SP1C	SP2A	SP2B	SP2C	SP2D	SP2E
Chlorophyll	X	X	X	X	X			X		X	X		X	
One carotenoid	X	X	X			X			X		X		X	
Two or more carotenoids								X		X		X		
Scytonemin						X	X						X	
C-Phycocyanin												X		
Compound 1		X	X	X										
Compound 2									X					
Calcium oxalate monohydrate Whewellite	X			X	X									
Calcium oxalate dihydrate Weddellite	X													
Hematite	X		X	X		X				X				X
Limonite/goethite										X				
Calcite						X								X
Hydrocerussite						X								
Gypsum						X								
Pyrophyllite			X											
Rutile													X?	X?
	**SP3A**	**SP3B**	**SP4A**	**SP4B**	**SP5A**	**SP5B**	**SP6A**	**SP7A**	**SP7B**	**SP** **8A**	**SP9A**	**SP10A**	**SP10B**	**SP11A**
Chlorophyll		X	X	X			X	X	X	X		X	X	X
One carotenoid	X	X	X		X		X	X	X		X			
Two or more carotenoids												X	X	X
Scytonemin			X			X	X	X						
C-Phycocyanin					X								X	
Atranorin or parietin				X										
Cholesterol					X									
Parietien									X					
Compound 3							X							
Calcium oxalate monohydrate Whewellite				X										
Hematite					X									
Calcite					X									
Aragonite	X						X							
Quartz									X					
Realgar											X			
	**SP12A**	**SP12B**	**SP12C**	**SP12D**	**SP13A**	**SP13B**	**SP14A**	**SP14B**	**SP14C**	**SP15A**	**SP15B**	**SP15C**	**SP16A**	**SP17A**
Chlorophyll	X	X	X		X	X	X			X		X	X	
One carotenoid	X	X			X	X	X	X			X	X		X
Two or more carotenoids			X										X	
Scytonemin	X	X	X		X							X	X	
C-Phycocyanin								X					X	
Atranorin						X								
Phycobiliprotein			X											
Proteins							X							
Cellulose								X						
Compound 4				X										
Compound 5								X						
Compound 6								X						
Compound 7											X			
Compound 8													X	
Calcium oxalate monohydrate Whewellite							X	X	X					
Calcium oxalate dihydrate Weddellite							X	X						
Calcite								X						
	**SP18A**	**SP18B**	**SP18C**											
Chlorophyll		X												
Scytonemin			X											
C-Phycocyanin														
Compound 9	X													

When a rock is not porous, such as applite or basalt in Table 1, extremophiles must colonize the surface, or inhabit a crack, become located beneath translucent light colored minerals or inside a vacuole connected with the exterior by a pore (geo-strategies). In this last case, gas and water interchange can occur through the pore, and light can also reach the organisms through it. However, very few chasmoliths have been studied by Raman spectroscopy; hence, only seven specimens from the forty-five described in this review are chasmolithic in origin. Organisms living on the surface of a rock (epilith) are more exposed to the hazardous environmental conditions and they therefore require the operation of supplementary bio-strategies, since they are denied the useful protection offered by rock substrates. In these cases, some microorganisms produce one or more additional protective pigments. 

Extremophilic microorganisms living as biofilms, bacterial mats (microorganim sheets formed by organic compounds, bacteria, archaea and minerals mainly), in or on ice or snow, limestone and other sedimentary rocks have been studied using Raman espectroscopy, Table 1, but rather more analyses have been carried out on salt crystals, particularly halite and gypsum.

Most of the specimens analyzed by Raman spectroscopy originated from cold deserts, Table 2, from the Arctic and Antarctic regions, followed by those which came from hot deserts, which are, in most of the cases, related to halotrophic environments. Primary colonizations are also considered extremophilic because of the nutrient shortage. Despite this, specimens SERS2B, SERS2C and SERS2D were collected under cold climate conditions, with the limiting parameter being the high temperature, since they are all related with a hot spring environment.

From Table 1, it can be inferred that deserts, either cold or hot, have been the main environments studied. The current search for life in extra-terrestrial environments is currently focused on the planet Mars and on the planetary satellites Europa and Titan [77,78,79,80]. As water is undoubtedly the determining factor for life, the search for extraterrestrial life is focused on regions where liquid water could have existed. Nowadays, it is generally accepted that liquid water existed in the past on Mars, but this water disappeared in its ancient geological history into the subsurface or evaporated into space—although there is clear evidence recently for subsurface fluvial activity and water of crystallization tied up in mineral occlusions. Now, Mars is a cold and dry planet (at least on the surface) but, if life ever existed on Mars, it should have adapted to the ever more extreme environmental conditions assuming that it had the time to do so before the prevailing climate became too inhospitable. It is reasonable to assume that extremophilic microorganisms and their analogues are the most probable source if there is any chance of finding life signals on Mars, either extinct or extant. 

Sandstone, carbonated and saline rocks are the most investigated terrestrial substrata with biological colonization, Table 1. These sedimentary rock groups are widely spread on the Earth’s surface and have also been described on Mars. Although it is an advantage that water does not interfere with the Raman signals from extremophilic colonies and optimal results can be obtained from the study of microorganisms living in water, ice or snow, surprisingly few studies have been carried out on these systems.

Biomolecules and related minerals produced by the specimens described here have been summarized in Table 2a,b. In several extremophilic specimens, some kind of mineralogical change or mineral mobilization is observed, such as hematite accumulation on the rock surface, a deficiency of hematite noted around the organic colony, iron oxide transformation closely related to the microorganisms and changes between calcium carbonate phases (calcite to aragonite) where the organisms are detected; these alterations, after identification by Raman spectroscopy, can then be ascribed to a geo-strategic change. Hence, in these cases, minerals have also been included in the Table 2, but it is important to note that the aim of most of the investigations carried out and published has been focused only on microorganisms and their biomolecules, and not on the study of minerals, so naturally, the mineral occurrence has not been generally described.

Minerals related with extremophiles have also in some cases been deposited from surrounding areas by wind and rain and thereby become associated with the biological colony (SP7B). Other specimens show a clear relationship between aragonite and the microorganism area, such as in (SP3A), where calcite appears as a white dust in the crack surface and only in the immediate area of the colony does aragonite occur. A similar phenomenon happens in SP6A, where in a hydromagnesite rock, aragonite is present in the vicinity of the microorganisms. SP14B and SP14C show evidence of a calcium carbonate phase related with some of the microorganism layers, in spite of the fact that the rock substratum is, in both cases, a sandstone. An interesting case is the specimen RW, from Antarctica, where microorganisms colonize pores a few millimeters below the surface, but a layer of calcite, hydrocerussite and hematite appears, not in the organic layer itself, as in the previous examples, but just surrounding them; since hydrocerussite is a lead carbonate and lead is a toxic element, it was suggested that this mineral could play a role in the prevention of biological attacks upon the colony by predators. All these are examples of geo-strategic biological colonization.

Rutile appears in SP2D and E specimens, both in a sandstone substratum in the vicinity of the colonization zone; despite its nonoccurrence in the bulk of the rock, it is not possible to conclude that there is a biological transformation between anatase (a rutile polymorph) and rutile, nor that there is a direct relationship between its presence and the biological activity. Hematite appears in some of the sandstones described, giving a reddish-orange color to the rock; it is remarkable that microorganisms seem to have mobilized this mineral, creating a lighter colored area around them and concentrating the mineral on the surface of the rock, such as in ANT1, ANT5, EN1, SP2A or SP5A. It is interesting that, to our knowledge, there has never been a description of the presence of hematite in the colonized area, and rather when an iron oxide appears directly related with the microorganisms it is in the form of a goethite phase, such as in SP2A.

Chlorophyll and carotenoids are the most prevalent biomolecules found in the Raman spectra of extremophiles. However, in many papers, the precise Raman identification of the type of carotenoid has not been assigned specifically—a possible explanation for this is that the unambiguous identification of a carotenoid can be very difficult in natural samples because of several factors which operate in complex mixtures and which cause quite large discrepancies to occur between the observed carotenoid bands and their pure isolated counterparts [81]. What can be assessed from the Raman spectrum is the presence of one or more carotenoids in a system and this is well documented. Carotenoids play very important roles as protective pigments: As DNA repair agents, free-radical quenching molecules and UV-radiation screens (bio-strategies). Production of c-phycocyanin (bio-strategy), a light harvesting pigment, was mainly found in those extremophiles that have colonized dark rocks, such as in SP10B, or deep areas within the rock (SP2C, SP5A, SP14B, SP16A); other organisms with c-phycocyanin but no data on the rock color or depth are provided for the specimens SERS2C, SERS2D. Another frequently found pigment is scytonemin, which has been demonstrated to play an important role as a UV screening extracellular compound. 

The characteristic Raman signatures for each biomolecule and mineral identified for each extremophile described in this review are shown in Table 3.

There are some Raman bands observed which as yet are not assigned, but these have been cited in Table 2, Table 3 as Compounds 1 to 9. In most cases, no indication of a possible source was supplied, except for Compound 1 (a chlorophyll-like compound) and Compound 7 (an aromatic polyphenolic compound) but for Compounds 5 (aliphatic) and 6 (aromatic) only a rather vague idea is given.

**Table 3:** Raman characteristic signatures of the biomolecules and minerals related with extremophile microorganisms.

COMPOUNDS	RAMAN SIGNATURES
Chlorophyll	1326, 1285, 987, 916, 755, 744, 516
Carotenoids	1550–1500, 1145–1160, 990–1015
Scytonemin	1591, 1553, 1432, 1381, 1321, 1170, 984, 752, 675, 574
C-Phycocyanin	1638, 1582, 1463, 1369, 1272, 815
Atranorin or parietin (antraquinone)	1630, 963
Cholesterol	1445, 1437, 875, 539
Atranorin	1666, 1658, 1303, 1294, 1266, 588
Parietien	1671, 1613, 1553, 1387, 1370, 1277, 1255, 926, 458
Phycobiliprotein	1638, 1586, 1468, 1369, 1281, 1236, 1049, 665
Proteins	1659 (amide I)1240-1290 (amideIII)
Cellulose	1380,1292
Compound 1 chlorophyll-like	1637, 1568, 1480, 1407, 1343, 1320, 789, 688, 554, 499
Compound 2	1449, 1342, 952, 834, 748, 680, 595, 438, 257, 173
Compound 3	1468, 1439, 948, 884
Compound 4	1531, 1452, 1342, 1143, 748, 681
Compound 5 (aliphatic)	940
Compound 6 (aromatic)	1005
Compound 7 aromatic polyphenolic compound	1442, 1629, 1757
Compound 8	1544, 1500, 1436, 1356, 1316, 1306, 1154
Compound 9	1690, 1645, 1520, 1441, 1250
Calcium oxalate monohydrate (whewellite)	1490, 1463, 896, 504
Calcium oxalate dihydrate (weddellite)	1475, 910, 506
Hematite	610, 405, 292, 223
Limonite/goethite	555, 395, 299, 203
Calcite	1086, 713, 282, 156
Aragonite	1086, 708, 203, 156
Hydrocerussite	1051, 681, 412
Gypsum	1132, 1008, 679, 618, 492, 413
Pyrophyllite	813, 705, 353, 259, 214, 195, 171
Rutile	609, 442
Quartz	463, 205, 128
Realgar	342, 220, 192, 182

## 3. Conclusions

Raman spectroscopy has been demonstrated to be an appropriate technique for the study of extremophilic microorganisms owing to its capabilities, particularly because it is a non-destructive technique, involving no chemical or textural structural changes in the sample and being able to identify organic and mineral compounds in admixture without any physical or chemical sample preparation.

From the specimens studied using this technique, it is possible to conclude that particular biomolecules are generally not related to any specific protective strategy apart from phycocyanins involved in photochemical light enhancement collection; chlorophyll and carotene are the most extensively occurring pigments and can be relatively easily detected using Raman spectroscopy. However, only extremophiles from cold and hot deserts have been most widely studied followed by halophiles and hot spring organisms; with such few studies having been undertaken overall, we can conclude that there are not enough data as yet to make unequivocal conclusions about the existence of common extremophilic survival strategies related to climate or substratum.

Raman spectroscopy is now vitally important as an analytical tool for the search for life on Mars and for related space missions, since it can primarily detect organic and inorganic compounds in admixture without their separation being effected; at the same time, it is important to create a large database of terrestrial extremophiles from as diverse a range of possible hostile environments as possible and to indentify the survival bio- and geo-strategies to provide an understanding of the key biomolecular protection chemicals operative prior to the exploration of planetary surfaces; then it should be possible to devise schemes to understand their strategic methodologies and mechanisms for survival; in this, Raman spectroscopic analysis will occupy a crucial role.
